# A water-based green approach to large-scale production of aqueous compatible graphene nanoplatelets

**DOI:** 10.1038/s41598-018-23859-5

**Published:** 2018-04-03

**Authors:** Ji-Heng Ding, Hong-Ran Zhao, Hai-Bin Yu

**Affiliations:** Key Laboratory of Marine Materials and Related Technologies, Key Laboratory of Marine Materials and Protective Technologies of Zhejiang Province, Ningbo Institute of Materials Technology and Engineering, Chinese Academy of Sciences, Ningbo, 315201 P. R. China

## Abstract

The unique properties of graphene are highly desired for printing electronics, coatings, energy storage, separation membranes, biomedicine, and composites. However, the high efficiency exfoliation of graphene into single- or few-layered nanoplates remains a grand challenge and becomes the bottleneck in essential studies and applications of graphene. Here, we report a scalable and green method to exfoliate graphene nanoplatelets (GNPs) from nature graphite in pure water without using any chemicals or surfactants. The essence of this strategy lies in the facile liquid exfoliation route with the assistance of vapor pretreatment for the preparation of edge hydroxylated graphene. The produced graphene consisted primarily of fewer than ten atomic layers. Such the water soluble graphene can be stored in the form of dispersion (~0.55 g L^−1^) or filter cake for more than 6 months without the risk of re-stacking. This method paves the way for the environmentally friendly and cost-effective production of graphene-based materials.

## Introduction

Following the discovery of graphene-based materials, graphene has been applied vigorously in fields ranging from electronics to medicine recent years because of its attractive electrical, mechanical, optical and chemical properties^1–3^. However, the production of true (single-layer) graphene in bulk quantity in liquid form graphite dispersions has been an unreachable goal for materials scientists in the past decade^4,5^. It was known that several routes have been explored to produce graphene which include micromechanical cleavage, topological growth on metal and non-metal substrate, and chemical oxidation of graphite^6,7^. It is also evident that the three approaches suffer from scalability, cost effectiveness and quality issues. Liquid-phase exfoliation is a promising approach to realize scalable production of high-quality graphene or other layered materials^8^. Generally, graphite can be exfoliated in aqueous or organic solutions with a surfactant by using mechanical cleavage approach (e.g., ultrasonic agitation^5^, ball-milling^9^, or mechanical shearing)^10^. During the processes for purification and dispersion, the exfoliation method suffers from the need for large quantities of solvents, which are usually expensive and need special care when handling. Despite having many impressive features, to date, graphene nanoplatelets can be dispersed in pure water only with a concentration less than 0.01 g L^−1^ and there is only slightly improved by adding dispersants like surfactants, ionic liquids^11^, superacids^12^, even long time sonication^13^. On the other hand, long time storage or reducing the amount of solvent destabilizes the dispersion and leads to the re-stacking of nanoplatelets. This means that an enormous amount of solvent is needed and only a small amount of graphene is produced, which is economically infeasible and environmentally unfriendly^14–18^.

Some new approaches for scalable production of graphene nanoplatelets (GNPs) were recently reported, which include ball-milling graphite in the presence of some chemical reactants. For example, Zhang *et al*.^19^ found a novel ball-milling exfoliation procedure with the addition of modifier of DY50 during the milling process to produce graphene nanoplatelets with high production yield (~100%). Salvatierra *et al*.^20^ reported a simple and novel route, based on a reaction between the aqueous acid solution and the magnesium to provide a large amount of hydrogen gas and heat to enhance production yield of graphene. According to these results, high-speed ball-milling generates kinetic energy sufficient to break C-C bonds, as well as π-π bonds between the graphite layers. However, these methods are commonly time-consuming, comprising multiple steps in hazardous conditions. Also, the proposed approaches were mostly resulted in the functionalized multi-layered GNPs that need further treatment to reach the pure few layered graphene^21,22^. Dong *et al*.^23^ present an fully-chemical-free method for pretreatment, on mild oxidation of graphite exfoliation and dispersion of graphene in water, which can be stored in the form of a concentrated slurry (50 g L^−1^) or filter cake for months without the risk of re-stacking. Bepete *et al*.^4^ achieved single-layer graphene stable dispersion in degassed pure water with a concentration of 0.16 g L^−1^_._

In this paper, we present a facile and eco-friendly method to exfoliate graphite in a layer-by-layer way to produce GNPs in pure water. In contrast to the conventional exfoliations in a solvent dispersed system, we strive for an approach in which GNPs are produced and stored as flocculated aqueous solution with a high concentration. In this typical process, natural graphite powder was added to distilled water at a mass fraction of 10%, followed by sonication the mixture into a homogeneous form. The exfoliation process involves the following sequences: high temperature vapor leads to the curling and delamination of the graphite, insertion of water molecule along the fissure, cutting by the reaction of graphite edge and water molecule, or direct peeling off from the parent materials. The yield of ultrathin graphene is about 23.5% by weight, and the concentration of graphene in pure water is as high as 0.55 g L^−1^. This process is environmentally friendly since thermal energy is readily available and water is a nontoxic medium, leaving no impurities in the product.

## Experimental Section

### Materials

Natural graphite powder (natural, ~325 meshes, 99.8% metal basis) and ethanol (95%) were purchased from Sigma-Aldrich. Distilled water was subjected to agitation with a rotating Teflon stirrer under a reduced pressure of 0.1 mbar for 40 min to obtain degassed water. All the other reagents were used as received.

### Method

The exfoliation of graphite was following by two steps. Firstly, 5 g of graphite powder was added into 50 mL degassed water. The mixture was subjected to sonication at ~500 W (75% of total power) for 30 min by using a tip sonicator (SJIA 650 W), resulting in a black graphite slurry. The mixture was further transferred to a stainless steel autoclave and kept at 150 °C for 2 h with a 1500 rpm mechanical stirring. After cooling down to room temperature, the black slurry was collectedfrom the autoclave and diluted by adding 500 mL distilled water. Subsequently, the solution was sonicated for 20 min at ~500 W using a tip sonicator, and centrifuged at 3000 rpm for 30 min to remove other unreacted materials. The as-obtained sample was dried at 60 °C in a vacuum oven for 24 h.

### Characterization

The following equipment was used: Scanning electron microscope (SEM) images were acquired using a FEI QUANTUM 250FEG field emission scanning electron microscope. The SEM samples were prepared by placing a small amount of as-obtained solid on the conductive carbon adhesive, which was stuck to an aluminum sample holder. Transmission electron microscope (TEM) images were observed on a high-resolution JEOL JEM-2100 transmission electron microscope instruments at an acceleration voltage of 200 kV. Atomic force microscope (AFM) images were obtained measurements with a Digital Instruments Dimension 3100 instrument using Si/SiO_2_ substrate with the graphene ethanol suspension deposited on it. X-ray photoelectron spectroscopy (XPS) (AXIS ULTRA DLD) was applied to the confirmation of the chemical composition. Fourier transform infrared spectrometer (FTIR, THERMO Nicolet 6700) was used to confirm the structure of graphene. Raman spectroscopy was performed to confirm the quality of the graphene nanosheets exfoliated in water and compare them with the raw materials. The thermal behavior was performed with a TG-209 F3 instrument (NETZSCH, Germany) at a heating rate of 10 °C min^−1^ from room temperature to 800 °C under nitrogen atmosphere. Contact angle measurements were carried out by a contact angle goniometer (CAM101, KSV) and the results were presented as the average of three measurements at different locations. UV-vis absorption spectra were recorded on a UV-2550 spectrophotometer with the wavelength ranging from 200 nm to 800 nm. Electrical conductivity was measured from current-voltage curves under small currents that do not generate significant self-heating.

## Results and Discussion

**Figure 1:**
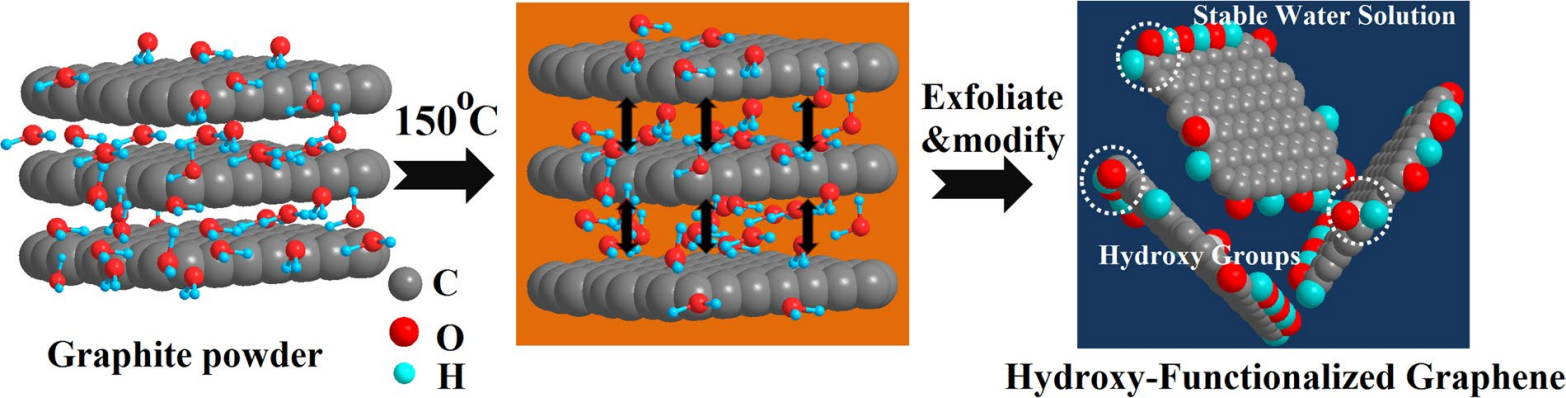
Schematic illustration of the exfoliation process.

In this research, we used bulk-layered graphite material to obtain atomically thin graphene platelets in pure water Fig. 1), and such the synthesis of a stable graphene aqueous dispersion was beautifully simple and efficient. While the mechanism of graphene exfoliation via this method has yet to be fully understood, we believe that a combination of sonication, high temperature, and the water vapor play a dominant role in deciphering the exfoliation path. The final yield of atomically thin graphene platelets is about 23.5% by weight in our work. Note that the precipitate can be recycled for the next run of the exfoliation as mentioned above, thus we believe that raw graphite could be fully exfoliated into graphene through several cyclic experiments. It is well known that due to its large aspect ratio and strong Van der Wals forces between platelets, graphene easily forms sediments in water solution when exceeding the limit concentration of 0.01 g L^−1^. Although the dispersibility of graphene can be improved in the present of surfactant or special ionic liquids in water, higher viscosity and limited exfoliation efficiency make the large-scale production of GNPs impractical. In contrast, our graphene can be dispersed in pure water with a concentration of 0.55 g L^−1^ with remaining stable more than months in room temperature, which is much higher than most previous reports (usually <0.16 g L^−1^)^4,24^. As the result, this affords opportunities for a high concentration and low-cost as well as massive scale production of GNPs.

**Figure 2:**
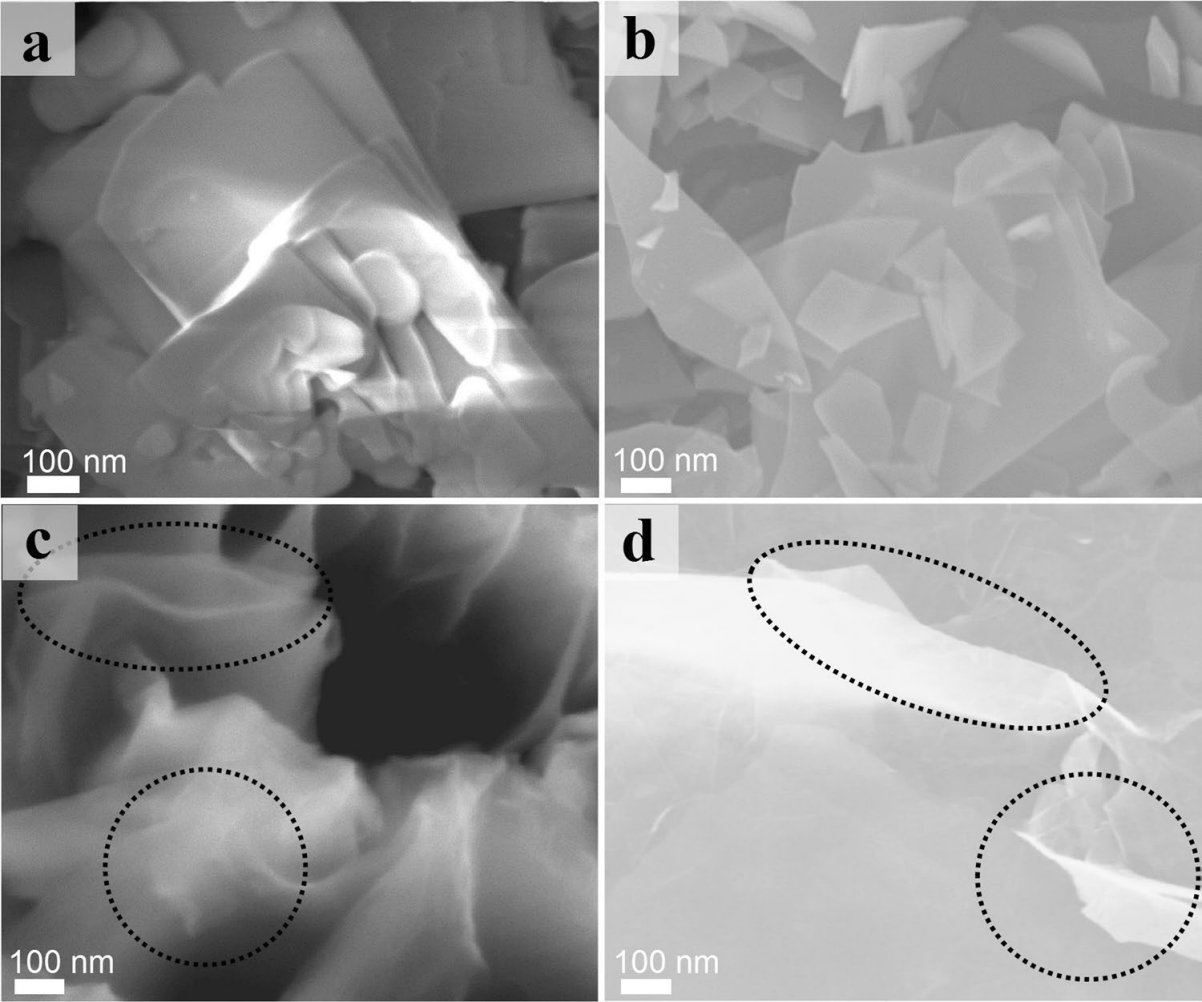
SEM image of graphite (**a**), a very flat GNPs (**b**), and typical nanoscrolls (circled in black) (**c**) and (**d**).

Once graphene could be exfoliated, the morphology of GNPs was an important parameter for further application or characterization. In order to study the morphology change after the exfoliation, scanning electron microscopy (SEM) images of the parent initial graphite powder and GNPs were observed and the results were shown in Fig. 2. Compared with the initial graphite (Fig. 2a), the GNPs display a much smaller micron-sized and few-layer randomly overlapped flat nanosheet-like morphology (Fig. 2b). As we can see that these GNPs are almost entirely transparent, presenting their ultrathin nature. The size of folded platelets is ranging from several hundred nanometers to several micrometers, which is similar to those in previous reports^25–27^. In addition, it can be seen from Fig. 2c and d that GNPs with nanoscroll morphologies, which is strikingly similar with exfoliated hexagonal boron nitride reported before by Qian, *et al*.^7^. This phenomenon demonstrates that the flexibility of platelets is high and the thickness of the peeled-off sheets is small. Due to the novel scroll topology, we believe these platelets may have different properties from common carbon nanoscrolls^28^.

**Figure 3:**
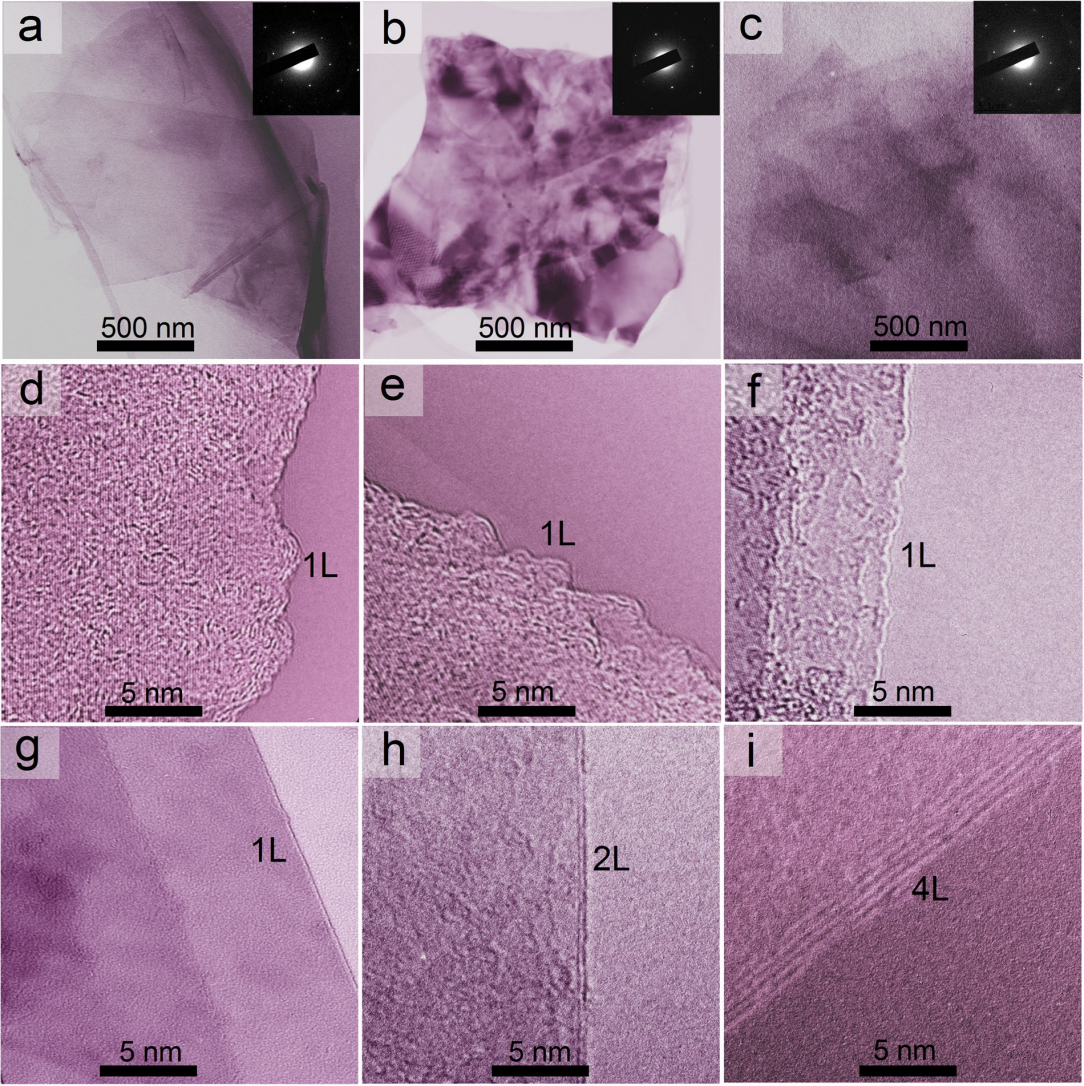
TEM image and the corresponding SAED pattern of single-layer GNPs (**a**–**c**), and HRTEM images of GNPs (**d**–**i**).

The morphology of GNPs was further analyzed by transmission electron microscopy (TEM). Samples for TEM were prepared by drying a droplet of the graphene suspension on a mica substrate. TEM images were obtained using a JEM-2100F high-resolution transmission electron microscope at an acceleration voltage of 200 kV. Figure 3a–c show that the GNPs have an ultrathin, transparent and overlapped flat structure, which provides clear evidences that the graphite are exfoliated thoroughly into very thin layers. Meanwhile, the sizes of these nanoplatelets determined by observing low-resolution TEM images are in good agreement with the SEM results. To further probe the thickness and the structural information of the as-made GNPs, we also present the selected area electron diffraction (SAED) patterns for all the samples (insets of Fig. 3a–c). The SAED pattern images reveal the isolated single-layer GNPs with the typical hexagonal symmetry^29–31^. GNPs were further analyzed through high-resolution TEM (HR-TEM) and these images were listed in Fig. 3d–i. As determined from these figures, these nanoplatelets observed mostly consist of 1–4 atomic layers.

**Figure 4:**
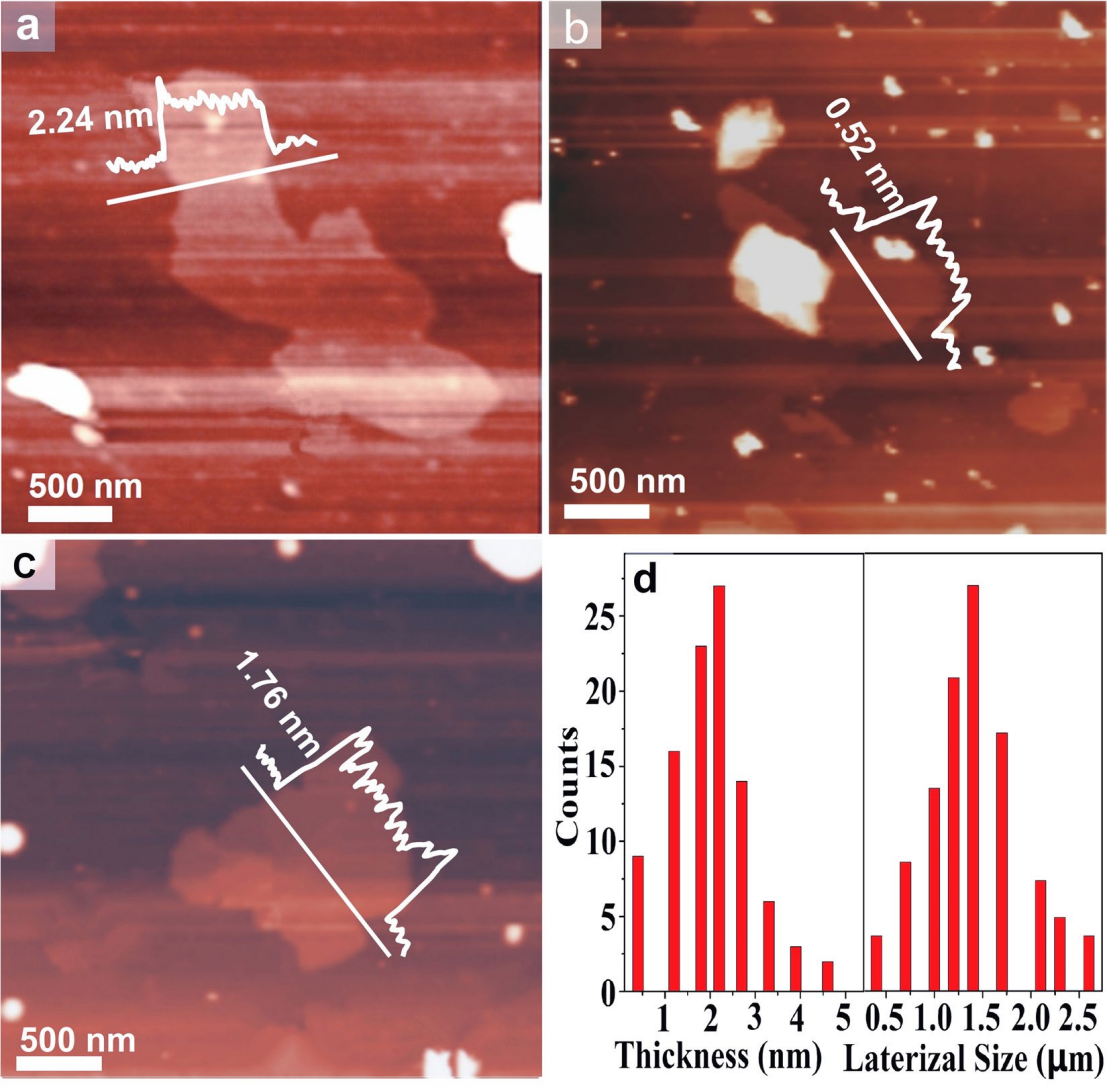
Representative AFM images of GNPs (**a**–**c**), and the distribution of the lateral size andthickness of GNPs (**b**).

The thickness and fine structure of GNPs were further measured on an Atomic force microscope (AFM). The samples used for AFM tests were prepared by depositing the corresponding dispersions on Lacey carbon grid and dried under vacuum at 50 °C for 12 h. As shown in Fig. 4a–c, the typical AFM images indicate that the thickness of platelets are about 2.24 nm, 0.52 nm and 1.76 nm, which corresponding to six-layers, single-layer, and four-layer GNPs, respectively. As shown in Fig. 4d, the statistical analysis of over 100 flakes displays that >25% of the GNPs are single layer (<1 nm in thickness) with a lateral size ranging from 0.5 to 2.5 μm. Intriguingly, there are no more than 10 layers of GNPs found in the observed area. The above mentioned SEM and TEM results are confirmed by AFM observations. Both TEM and AFM results suggested that the GNPs as-prepared were predominantly few-layered nanoplatelets, consistent with the expectation that the treatment of graphite with water vapor resulted in the exfoliation and peeling into platelets from the precursor graphite.

**Figure 5:**
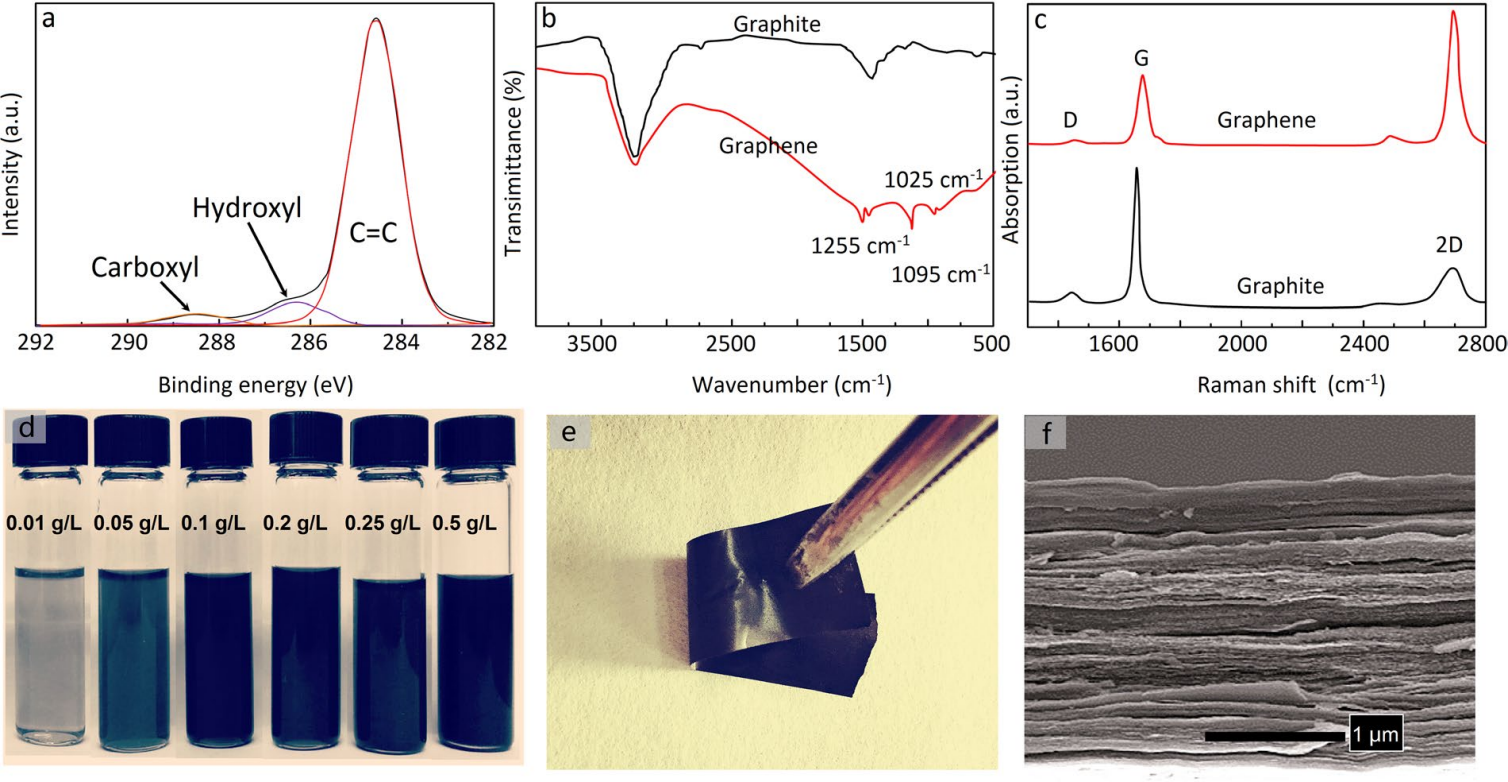
Characterization of the chemical compositions of GNPs: (**a**) XPS, (**b**) FTIR, and (**c**) Raman spectroscopy; (**d**) photographs of solutions of graphene dispersed in deionized water for one month, (**e**) photograph of a 3 µm thick graphene paper (1 × 3 cm), and (**f**) SEM side view image of a thick graphene paper.

In order to further characterize the crystalline structure and composition of as-obtained GNPs, we used X-ray photoelectron spectroscopy (XPS), Fourier transform infrared spectroscopy (FTIR), and Raman spectroscopy. XPS spectrum of powder samples is shown in Fig. 5a. The result reveals that high-temperature vapor exfoliation leads to successful functionalization of hydroxyl (286.3 eV) and carboxyl (288.8 eV) hydrophilic groups on graphene^32,33^. We have carried out detailed FTIR investigation to monitor the evolution of the functional groups in graphene samples during the exfoliation process. Figure 5b displays the FTIR spectra of GNPs as-prepared. For the exfoliated graphene sample, the new absorption peaks at 1025 and 1095 cm^−1^ are assigned to stretching vibrations of C-O in hydroxyl, and the sharp absorption peak at 1255 cm^−1^ belongs to stretching vibrations of C-O in carboxyl groups, while they were not observed in the FTIR spectra of initial graphite sample. Raman spectroscopy has been used as a powerful tool to study graphene-based materials, to determine the number of layers, the stacking sequence in the case of multiple layers, doping, and the amount and nature of defects. Raman spectroscopy provides a non-destructive method to determine whether these functional groups of graphene are present on the surface, as in graphene oxide, or on the edges^34^. Generally, graphene possesses three main features in Raman spectroscopy, G-peak, D-peak and 2D-peak and every peak has different physical origins. The G-peak at about 1580 cm^−1^ and the D-peak at about 1350 cm^−1^ are attributed to the sp^2^-hybridiaed carbon bonds in the graphene lattice and the edge or defects in the lattice, respectively^18^. From Fig. 5c, the intensity of D-peak in the Raman spectra of the graphene sample is negligible, which implies the bare existence of functionalization or defects in the network. For the initial graphite, the Raman spectrum has similar shapes with the exfoliated graphene, which demonstrates the treatment of graphite with high temperature vapor likely broke C-C bonds and generated hydrophilic –OH and –COOH groups at the edges and defective sites rather than onto the graphene surface. The featured 2D-peak band 2690 cm^−1^ and the larger intensity ratio of the 2D-peak to the G-peak (*I*_2D_/*I*_G_) imply the few layer feature of graphene^18^. The amount of –OH and –COOH groups was also evaluated from the thermogravimetric analysis (TGA), which suggests ~2.26 wt% content of hydrophilic groups in the functionalized graphene edges (Supplementary Figure 1). The surface characteristics of graphite and graphene were tested by the measurement of contact angle of water on solid surface (Supplementary Figure 2). It can be observed that the GNPs as-prepared exhibit lower contact angle with an average value of 42 ± 1°, whereas the graphite has an average contact angle of 97 ± 1°. The decrease in contact angle was an evident of graphite exfoliation into graphene, implying the hydrophilic of exfoliated graphene improved due to the –OH and –COOH species produced^25^.

Owing to its high hydrophilicity and the thin lamellar structure, GNPs were easily dispersed in water. In addition, since cavitation is the key factor to the graphene dispersion in water, the particle dissolution yield can be improved by heating the water more and lowering the pressure, which enhances the level of cavitation. GNPs were re-dispersed in water at a concentration of 1 g L^−1^and centrifuged at 2000 rpm for 30 min, and the supernatant was collected as stable graphene dispersion. The concentration of aqueous GNPs dispersion was calculated to be ~0.62 g L^−1^ (Supplementary Figure 3). What’s more, a high stability was found about the graphene dispersion, with ~90% of the suspension presenting even after 1 month (concentration of ~0.55 g L^−1^), which is the highest at present report based on dispersion in pure water. Although stability seems to decrease over time in the case of room temperature storage, we observed that precipitation ceases completely after 30 days and that the solutions maintain their same colour for longer than six months. For example, the aqueous dispersions with different concentrations about 0.015~0.5 g L^−1^ were unchanged after stored for six months at room temperature (Fig. 5d). The graphene dispersion with a concentration of 0.05 g L^−1^ shows an absorption peak at ~268 nm in the UV-vis absorption spectrum, which is the exact wavelength previous reported for the absorption of few-layered GNPs (Supplementary Figure 4). In addition, the graphene solution exhibits a clear Tyndall effect based on the light scattered from large graphene platelets (inset of Supplementary Figure 3). As a result, due to the simple synthetic process the exfoliation of graphite into graphene dispersion can be carried out at large scale.

With the rapid development of the electronics industry, conductive paper has become increasingly critical in enhancing the performance and reliability of the devices. GNPs have been used as fillers in polymer composites to considerably enhance the electrical conductivity, which is particularly important for applications in electronic packaging. For the purpose of developing a conductive material, we propose to construct an ultrathin conductive film made of GNPs only. Therefore, the as-prepared GNPs were used as building blocks for the fabrication of a free-standing conductive paper by a simple filtration process. Graphene paper was prepared by filtrating 0.1 g L^−1^ aqueous graphene suspension (0.1–0.5 g L^−1^) with a PTFE membrane with size of 0.22 μm. These films can be easily peeled from the filter membrane. They tend to be shiny on the side which faced the membrane but matt black on the other side. It is well known that carriers must cross many inter-sheet junctions as they pass through the graphene films, however, which has been confirmed that such junctions limit the conductivity of graphene films. Thus, the graphene film was sandwiched between two thin and smooth polyimide films and pressed under a pressure of 30 MPa for 20 min to get a highly densed compress graphene film with densities of 1.75–1.85 g cm^−3^ (and so porosities of 20–25%). We expect that electrical conductivity of graphene film can be significantly improved by mechanical compression. As displayed in Fig. 5e, the graphene film shows a shiny metallic sheen. This is similar to previously reported films of reduced GO. The cross-sectional view images of the carbon film via SEM clearly implied the well-packed dense structures (Fig. 5f). The thickness of the film could be adjusted in the range 3~50 μm along with the different volume and concentration of graphene dispersion, and the conductivities of these films were further measured. These films were found to exhibit mean conductivity of 2.1 ± 0.1 × 10^4^ S m^−1^ before annealing and can be further improved to 4.8 × 10^4^ S m^−1^ after annealing at 500 °C for 2 h, which is much higher than the most of graphene films prepared by similar solution exfoliation^13–18,26–29,35,36^. Because the films were produced through a slow process, the annealing would be more ordered and decrease the content of oxygen-containing groups on graphene surface, which would result in the electrical conductivity enhancement. As a result, this level of conductivity is quite encouraging that the graphene films would be used as an electrode for electric devices.

## Conclusion

In summary, we present an effective and green method for large-scale synthesis of few-layered. This ability to use pure water to exfoliate and disperse 2D materials will enable not only cost-effective industrial and commercial applications but also facilitate research that requires water-based experiments. The results of our experiments imply that water vapor exfoliation at a high temperature results in the synthesis of graphene with good dispersion stabilities in water. As-prepared graphene can be highly dispersed in water, without any additives, at a concentration of 0.55 g L^−1^ with a shelf life of more than months at room temperature. This approach has several advantages including simple-step, low-cost, even without any use of expensive or sophisticated equipment. This remarkable feat results from the graphene-based layered materials. We have achieved graphene conductive film with water dispersed graphene material, which thus have significant potential for real-life applications.

## Electronic supplementary material

Supplementary Information
