# Supplementary material for: A water-based green approach to large-scale production of aqueous compatible graphene nanoplatelets
Source: Sci Rep. 2018 Apr 3;8:5567. doi: 10.1038/s41598-018-23859-5 (PMC5883015; doi:10.1038/s41598-018-23859-5)
Supplement: Supplementary file 1 — Supplementary Information [file 41598_2018_23859_MOESM1_ESM.doc]

**SUPPORTING INFORMATION**

**A water-based green approach to large-scale production of aqueous compatible graphene nanoplatelets**

Ji-Heng Ding*^1^, Hong-Ran Zhao^1^, Hai-Bin Yu*

*Key Laboratory of Marine Materials and Related Technologies, Zhejiang Key Laboratory of Marine Materials and Protective Technologies, Ningbo Institute of Materials Technology and Engineering, Chinese Academy of Sciences, Ningbo 315201, China.*

***^1^ These authors contributed equally to this work.***

****Corresponding author, Electronic mail: dingjh@nimte.ac.cn；haibinyu@nimte.ac.cn.***

**Experimental section**

It should point out that it is impossible to make sure every batch of commercially-obtained graphite powder is identical. Notably, the chemistry properties of graphene are very dependent on graphite sample preparation, purity, crystallinity, and microstructure. Because of this, the results of the experiment may be different from batch to batch.

**Characterization**


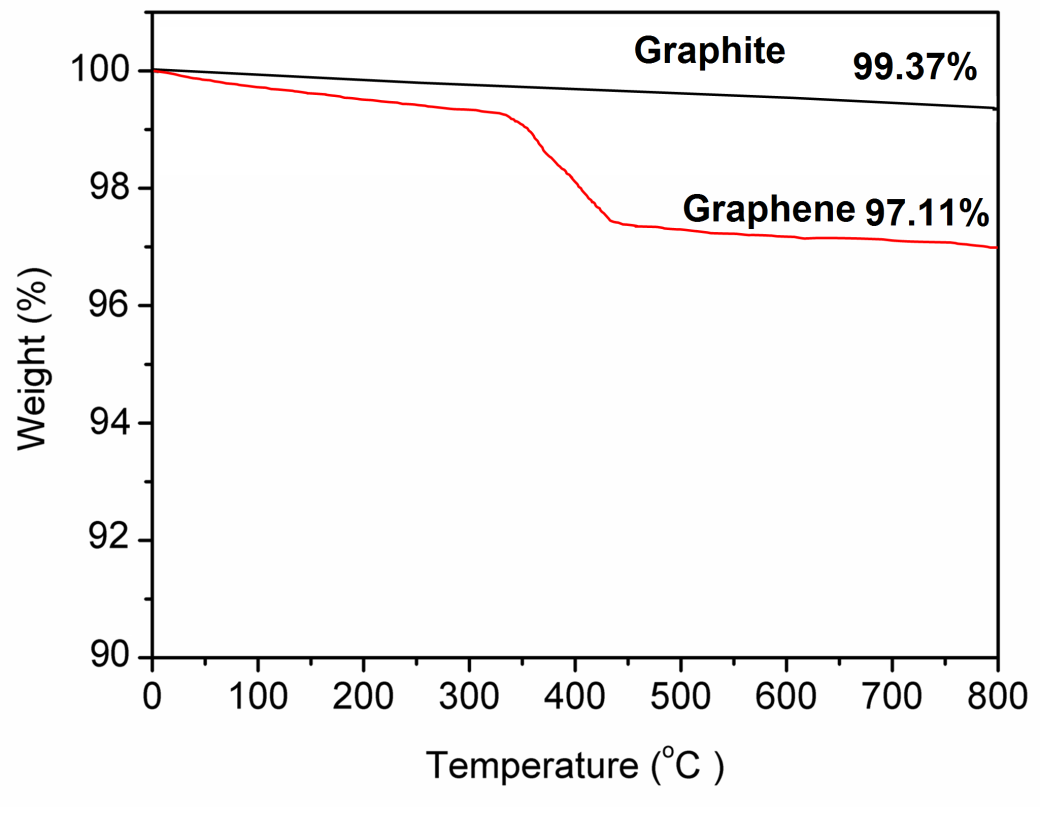


**Supplementary Figure 1.** TGA curves of graphite and few-layer GNPs.

**
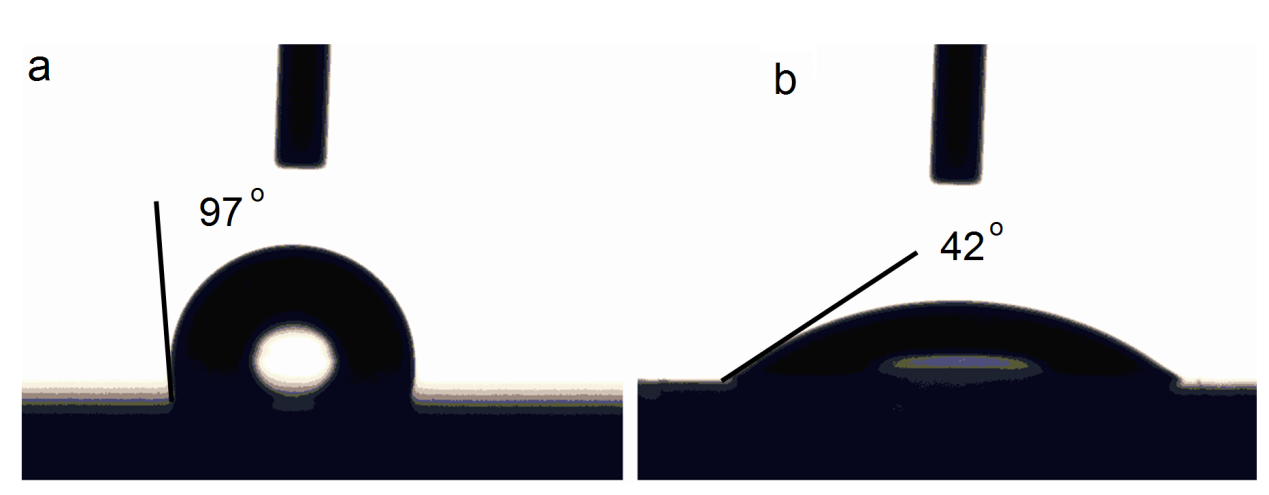
**

**Supplementary Figure 2.** Contact angle measurements. (a) graphite, and (b) few-layer GNPs.


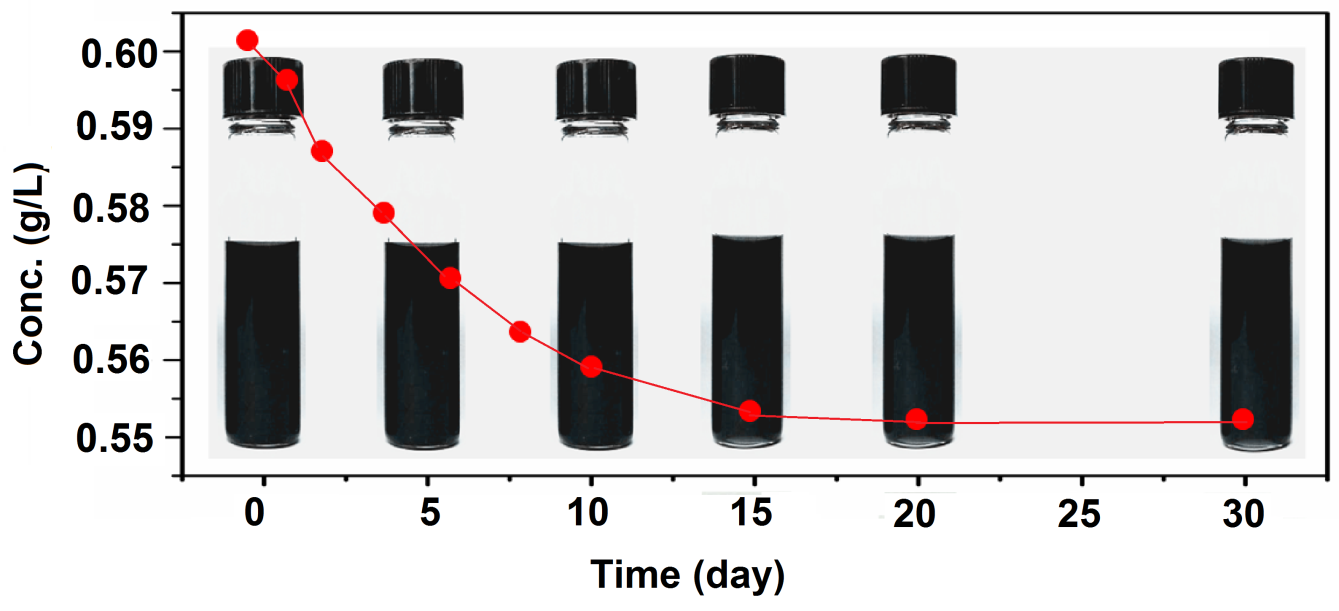


**Supplementary Figure 3.** Photographs of solutions of graphene dispersed in distilled water for one month.


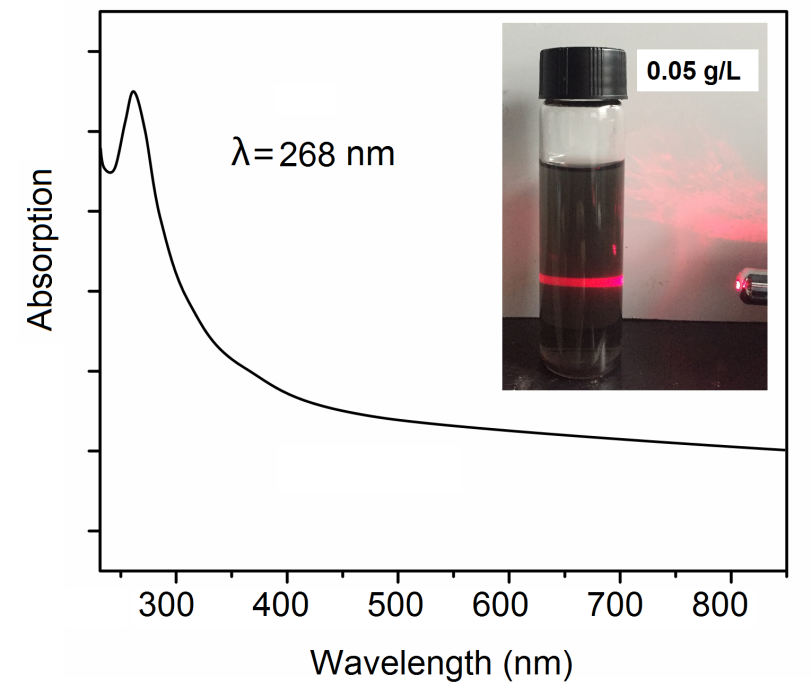


**Supplementary Figure 4.** UV-vis absorption spectrum shows an absorption peak at 268 nm .
